# A Case Series of Tuberculous Pericarditis: Clinical Profiles and Diagnostic Challenges

**DOI:** 10.7759/cureus.111822

**Published:** 2026-06-30

**Authors:** Ivneet Kour, Anand Singhal, Noorul Aysha, Varsha Gupta, Lipika Singhal

**Affiliations:** 1 Microbiology, Government Medical College &amp; Hospital (GMCH), Chandigarh, IND; 2 Internal Medicine, Maharishi Markandeshwar (MM) Institute of Medical Sciences &amp; Research, Mullana, IND

**Keywords:** ada, cbnaat, extrapulmonary tb, mycobacterium tuberculosis, tuberculous pericarditis

## Abstract

Tuberculous pericarditis (TBP) is an uncommon but serious manifestation of extrapulmonary tuberculosis, accounting for approximately 1-2% of extrapulmonary tuberculosis (TB) cases. Its diagnosis remains challenging because of the paucibacillary nature of the infection and the limited sensitivity of conventional diagnostic methods. Patients suspected of having tuberculous pericarditis between 2022 and 2024 were analyzed in this prospective case series. Various diagnostic tests were performed in suspected cases of tuberculous pericarditis, and the Cartridge-Based Nucleic Acid Amplification Test (CBNAAT) detected *Mycobacterium tuberculosis* in three samples. Clinical data, radiological findings, pericardial fluid characteristics, and microbiological test results were reviewed and analyzed for the patients with tuberculous pericarditis. Our study suggests that men in the middle to older age groups may be at higher risk. The most common clinical findings were fever, dyspnoea, and pericardial effusion. This study highlights the heterogeneous clinical and laboratory profile of TBP, which can occur in diverse patient populations, ranging from immunocompromised individuals to otherwise healthy adults.

## Introduction

Tuberculosis is an infectious disease caused by *Mycobacterium tuberculosis* (MTB). Although the lungs are the most commonly affected site, the bacilli can spread to almost any organ, including the pericardium, resulting in tuberculous pericarditis (TBP) [1,2]. According to the Global TB Report 2020, extrapulmonary tuberculosis (EPTB) accounted for 16% of the 7.1 million reported tuberculosis cases worldwide, while in India, EPTB constitutes approximately 20-24% of all tuberculosis cases across all age groups [3,4]. TBP, a relatively uncommon form of EPTB, accounts for about 1% of all TB cases and 1-2% of extrapulmonary TB cases [4-6]. Its diagnosis remains difficult due to the paucibacillary nature of the infection, diverse clinical presentations, and low sensitivity of conventional tests. Microscopy shows positivity rates of 0-42%, while culture demonstrates 53-75% sensitivity [7]. If untreated, TBP may lead to life-threatening complications such as cardiac tamponade, constrictive pericarditis, or death [5].

Cardiovascular tuberculosis predominantly affects the pericardium, with myocardial and other cardiac involvement being rare. While tuberculous pericarditis is more commonly observed in immunocompromised patients, especially those with HIV infection, it may also develop in individuals without underlying immunosuppression [8]. Despite advances in molecular diagnostics, published reports describing Cartridge-Based Nucleic Acid Amplification Test (CBNAAT)-confirmed tuberculous pericarditis remain limited, particularly from North India. Here, we report three CBNAAT-confirmed cases of tuberculous pericarditis managed at a tertiary care hospital in Chandigarh.

## Case presentation

Case 1

An intravenous drug user presented in February 2022 with a 15-day history of shortness of breath and bilateral leg swelling. On evaluation, the patient was found to have decompensated chronic liver disease and was diagnosed as hepatitis C virus (HCV) positive, following which direct-acting antiviral (DAA) therapy was initiated. On admission, SpO_2_ was 92%, BP was 80/60 mmHg, and jugular venous pressure (JVP) was elevated. He developed shock requiring intubation and two days of ventilation, then improved with conservative management.

Evaluation showed a large pericardial effusion, managed with a pleuro-pericardial window and pigtail drainage. Aerobic culture of the pericardial fluid revealed growth of *Escherichia coli*, which was resistant to amikacin, ampicillin, cefepime, cefotaxime, ceftazidime, ciprofloxacin, piperacillin-tazobactam, and trimethoprim-sulfamethoxazole. CBNAAT of pericardial fluid detected MTB with a low bacterial load, and the strain was sensitive to rifampicin. Biochemical examination of the pleural fluid showed an adenosine deaminase (ADA) level of 254 IU/L.

Serial hematological investigations showed microcytic hypochromic anaemia with low mean corpuscular volume (MCV) (76-78 fL) and low mean corpuscular haemoglobin (MCH) (24-25 pg). Episodes of neutrophilia (76-79%) and lymphopenia (9-13%) correlated with acute infectious and inflammatory responses during active tuberculosis. The patient was treated with intravenous piperacillin-tazobactam, anti-tubercular therapy, a DAA regimen (sofosbuvir + velpatasvir), and supportive medications including lactulose, calcium, vitamin D3, and insulin. The patient improved and was discharged in stable condition.

Case 2

A 61-year-old male farmer presented to the cardiology outpatient department in August 2023 with complaints of shortness of breath and orthopnoea for one week. He also reported a history of low-grade fever for the preceding month and unintentional weight loss. The patient was a chronic smoker with no prior history or contact with tuberculosis and was HIV-negative with no history of diabetes mellitus.

**Figure 1:**
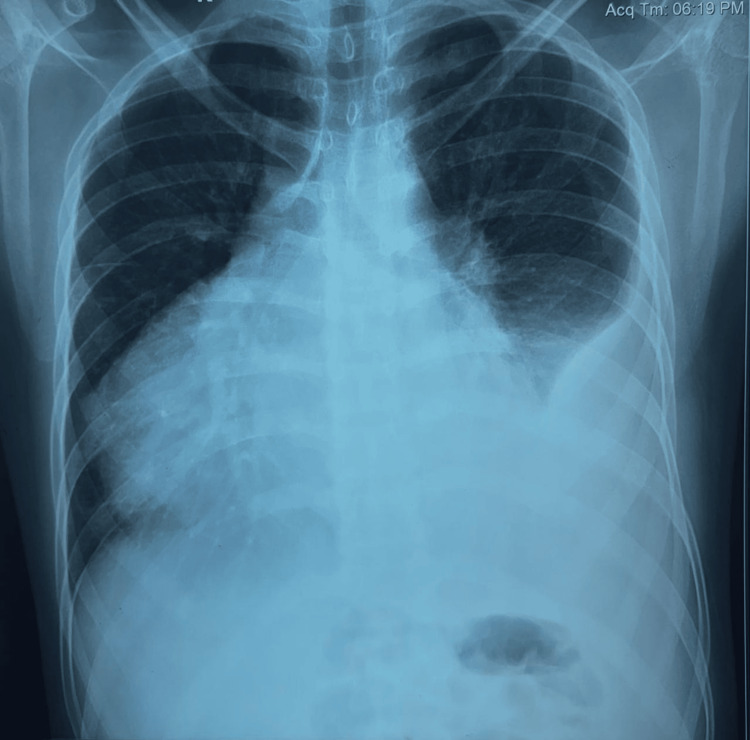
Chest radiography showing tuberculous pericardial effusion along with left-sided pleural effusion.

Two-dimensional echocardiography revealed a moderate pericardial effusion anterior to the right ventricle measuring 16 mm, along with mild tricuspid regurgitation. Chest radiography demonstrated bilateral pleural effusion, and pleural fluid analysis indicated a transudate with lymphocytic predominance (Figure 1).

**Figure 2:**
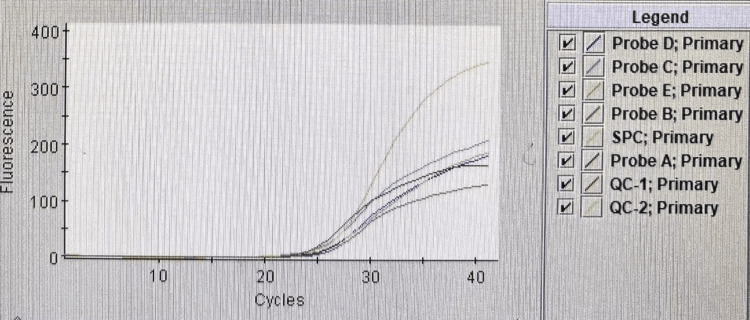
Xpert MTB/RIF assay output showing low-level detection of Mycobacterium tuberculosis (MTB) with no rifampicin resistance detected. MTB/RIF, Mycobacterium tuberculosis/rifampicin.

Contrast-enhanced CT (CECT) of the thorax showed a well-circumscribed pericardial effusion with a maximum thickness of 45 mm and features suggestive of impending cardiac tamponade. Pericardial fluid aspiration was performed, and the ADA level was elevated at 63 U/L. The initial CBNAAT was negative, while a repeat test performed the following day was positive with a low bacillary load (Figure 2).

Based on these findings, a diagnosis of TBP was established, and the patient was initiated on standard antitubercular therapy (ATT). Serial laboratory monitoring demonstrated clinical and biochemical improvement. On initial evaluation, hemoglobin was 9.7 g/dl, total leukocyte count 8,500/mm^3^, platelet count 2.82 × 10^5^/mm^3^, serum sodium 133 mmol/L, serum potassium 4.5 mmol/L, and alkaline phosphatase (ALP) 156 U/L. Follow-up evaluation three days later showed normalization of electrolytes and liver enzyme levels, with serum sodium 134 mmol/L, serum potassium 4.4 mmol/L, and ALP reduced to 123 U/L. These findings reflected a favorable therapeutic response with improvement in hepatic function and no evidence of ATT-induced hepatotoxicity or electrolyte imbalance. He was symptomatically better at the time of discharge.

Case 3

A 28-year-old female patient, a housewife, was admitted to the emergency ward in December 2024 with complaints of fever persisting for 20 days, accompanied by shortness of breath and orthopnoea. She had no prior history of tuberculosis, no known contact with tuberculosis cases, tested negative for HIV, was not immunocompromised, and had no history of diabetes mellitus. The patient reported significant weight loss, raising suspicion for an underlying chronic infectious or malignant process; however, malignant cytology of the pericardial fluid was negative, making a neoplastic etiology less likely and further supporting the diagnosis of tuberculous pericarditis.

**Figure 3:**
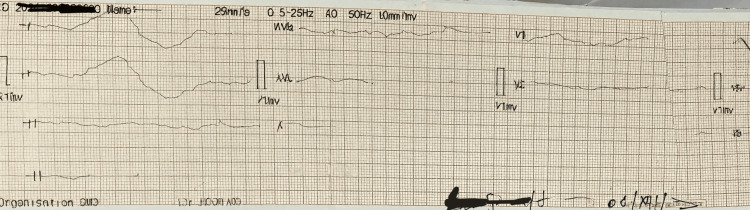
ECG low-voltage complexes suggestive of pericardial effusion.

Chest radiography revealed bilateral mild pleural effusion. ECG showed low-voltage complexes suggestive of pericardial effusion (Figure 3). Two-dimensional echocardiography initially demonstrated mild pericardial effusion, which later progressed to a large effusion.

**Figure 4:**
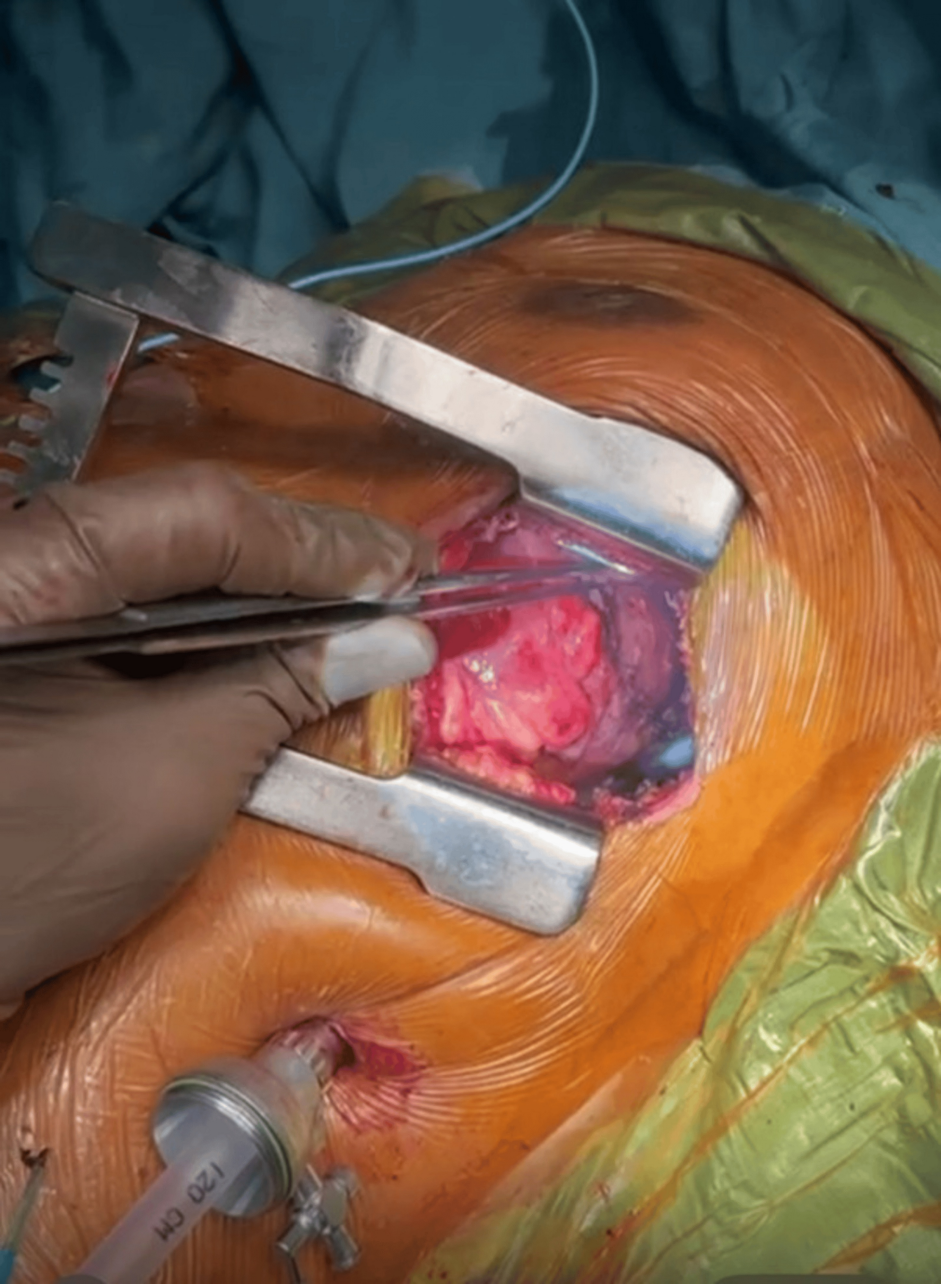
Pericardial effusion drainage (pericardiocentesis) showing aspiration of pericardial fluid for both therapeutic decompression and microbiological analysis.

Pericardial drainage was performed (Figure 4), and CBNAAT of the pericardial fluid detected MTB with a low bacillary load and rifampicin sensitivity, confirming a diagnosis of TBP.

Serial complete blood count (CBC) evaluations showed persistent anemia, with hemoglobin levels ranging between 8.6 and 9.9 g/dL and consistently reduced red blood cell counts (3.31-3.85 × 10^12^/L), suggestive of anemia of chronic disease associated with tuberculosis. The packed cell volume (PCV) remained below the normal range (28.4-37.3%), further corroborating the anemic profile. A mild to moderate leukocytosis was noted (total leukocyte count: 12.86-15.42 × 10^9^/L), predominantly neutrophilic (68-91%), indicative of an active inflammatory response. Early hematological findings included monocytosis (up to 10.8%), a feature frequently observed in tuberculosis.

The clinical presentation, lab parameters, and clinical outcomes of all the patients are listed in Table 1.

**Table 1:** Comparative summary of clinical presentation, diagnostic findings, and outcomes of the tuberculous pericarditis cases. ADA, adenosine deaminase; CBNAAT, Cartridge-Based Nucleic Acid Amplification Test.

Parameter	Case 1	Case 2	Case 3	Reference range
Presenting symptoms	Shortness of breath and bilateral leg swelling	Shortness of breath, fever, and weight loss	Shortness of breath, fever, and weight loss	-
Echocardiographic findings	A large pericardial effusion	A moderate pericardial effusion anterior to the right ventricle, with mild tricuspid regurgitation	Mild pericardial effusion, which later progressed to a large effusion	-
Hemoglobin (g/dl)	13.3	9.7	8.6-9.9	Male: 13-17; Female: 12-15
Total Leukocyte Count	2.79 × 10^9/L	8.5 × 10^9^/L	12.86-15.42 × 10^9^/L	4,000-11,000/mm^3^ (4-11 × 10^9^/L)
Neutrophils (%)	68.1	Not available	68-91	40-75
ADA pericardial fluid (IU/L)	244	63	Not available	<40
CBNAAT	Low bacterial load	Low bacterial load	Low bacterial load	-
Clinical outcome	Improved	Improved	Improved	-

## Discussion

Tuberculosis must be considered a potential cause of effusive pericarditis, particularly in individuals from endemic regions or those with predisposing conditions such as immunosuppressive or biologic therapy, HIV infection, diabetes mellitus, or substance abuse [5].

From January 2022 to December 2024, CBNAAT was performed on pericardial fluid samples from 22 patients with pericardial effusion to investigate suspected tuberculous pericarditis. The mean age of the study population was 44.45 years (range 3-78 years). Male subjects constituted the majority, accounting for 63.6% (14/22) of cases, while female subjects represented 36.4% (8/22). CBNAAT confirmed the presence of MTB in three patients, yielding an MTB positivity rate of 13.6%. According to a study by Shaik et al., tuberculosis was identified as the underlying cause in 17% of patients presenting with pericardial effusion [9].

In our study, male gender, middle to older age group, and environmental exposure appeared as potential risk factors in patients with tuberculous pericarditis. The predominant clinical features included fever, dyspnoea, and pericardial effusion, while progression to cardiac tamponade in one patient represented a rarer and severe manifestation.

Ziehl-Neelsen staining was negative in all patients, who exhibited a low bacillary burden with rifampicin sensitivity confirmed by CBNAAT, and none had a prior history of pulmonary tuberculosis. The diagnosis of TBP relies on microbiological and biochemical methods; however, it remains challenging due to the lack of a simple, rapid, and accessible test despite its significant morbidity and mortality [10].

In early disease, pericardial fluid shows neutrophil predominance, shifting to mononuclear cells in later stages. Diagnostic biomarkers such as ADA and interferon-gamma (IFN-γ) are useful adjuncts. Pericardial fluid culture remains the gold standard, while nucleic acid amplification tests like Xpert MTB/RIF (sensitivity 63.8%, specificity 100%) provide rapid confirmation [1].

Timely diagnosis through molecular assays such as CBNAAT, coupled with prompt initiation of anti-tubercular therapy, remains critical for achieving favorable outcomes and preventing life-threatening complications. The mortality associated with TBP remains high despite the initiation of anti-tuberculosis therapy, with an overall rate of 1.43 deaths per 100 person-months [7]. These cases highlight the varied clinical and laboratory characteristics of TBP, which can affect a wide range of patient populations, including immunocompromised people and otherwise healthy adults.

## Conclusions

Tuberculous pericarditis may present with diverse clinical manifestations, making diagnosis challenging. In this case series, conventional diagnostic methods like Ziehl-Neelsen staining yielded negative results, likely reflecting the low bacillary load associated with the disease. The combined use of clinical assessment, echocardiography, pericardial fluid analysis, CBNAAT, and ADA measurement supported the diagnosis and facilitated the timely initiation of anti-tubercular therapy. All three patients demonstrated favorable clinical outcomes during follow-up. However, these findings are based on a small case series and should be interpreted with caution. Larger studies are needed to further evaluate the diagnostic utility of these modalities and validate their role in the diagnosis and management of tuberculous pericarditis.
